# Advances in the Application of Transition-Metal Composite Nanozymes in the Field of Biomedicine

**DOI:** 10.3390/bios14010040

**Published:** 2024-01-12

**Authors:** Huixin Wang, Chunfang Cheng, Jingyu Zhao, Fangqin Han, Guanhui Zhao, Yong Zhang, Yaoguang Wang

**Affiliations:** 1 Shandong Provincial Key Laboratory of Molecular Engineering, School of Chemistry and Chemical Engineering, Qilu University of Technology (Shandong Academy of Sciences), Jinan 250353, China; wanghuixin2014@163.com (H.W.); ccf33012022@163.com (C.C.); zjyzjyoo@163.com (J.Z.); H15963929811@163.com (F.H.); 2 College of Chemistry and Chemical Engineering, Qilu Normal University, Jinan 250200, China; 3 Provincial Key Laboratory of Rural Energy Engineering in Yunnan, School of Energy and Environment Science, Yunnan Normal University, Kunming 650500, China; yongzhang7805@126.com

**Keywords:** nanozymes, composites, mimicking peroxidase, transition metal

## Abstract

Due to the limitation that natural peroxidase enzymes can only function in relatively mild environments, nanozymes have expanded the application of enzymology in the biological field by dint of their ability to maintain catalytic oxidative activity in relatively harsh environments. At the same time, the development of new and highly efficient composite nanozymes has been a challenge due to the limitations of monometallic particles in applications and the inherently poor enzyme-mimetic activity of composite nanozymes. The inherent enzyme-mimicking activity is due to Au, Ag, and Pt, along with other transition metals. Moreover, the nanomaterials exhibit excellent enzyme-mimicking activity when composited with other materials. Therefore, this paper focuses on composite nanozymes with simulated peroxidase activity that have been prepared using noble metals such as Au, Ag, and Pt and other transition metal nanoparticles in recent years. Their simulated enzymatic activity is utilized for biomedical applications such as glucose detection, cancer cell detection and tumor treatment, and antibacterial applications.

## 1. Introduction

In the natural world, enzymes are composed mostly of proteins and a small amount of RNA. There are seven enzyme classes as follows: transferases [1], oxidoreductases [2], hydrolases [3], lyases [4], translocases [5], isomerases [6], and ligases [7]. They play an indispensable role in various types of life activities, but it is difficult to expand the application of natural enzymes in other fields due to their harsher environmental requirements and their easy decomposition and inactivation in relatively harsh environments. Therefore, the development of an alternative to natural enzymes and materials with enzymatic activity has become an urgent problem to be solved. In recent years, nanomaterials, being a relatively new type of material, have a series of advantages, such as nanoscale size, an abundant porous structure for substrate and catalytic product transport, and a large number of active sites for catalysis, so they are undoubtedly the best choice as enzyme mimics [8,9]. Combining the advantages of nanomaterials and simulated enzyme properties, nanozymes have the features of convenient production, stable performance, and low cost [10,11]. At the same time, nanozymes address the requirements of poor stability of natural enzymes and harsher ambient temperature and pH, making them still active in harsh environments, such as the complex microenvironment in tumor cells [12], higher temperature in photothermal therapy [13], and intracellular hypoxic environment in photodynamic therapy [14]. Therefore, the development of stable and efficient nanozymes has become an urgent challenge for researchers to address.

At present, among the reported nanozymes, those mimicking oxidoreductases [15] occupy most of the reports, with more than 3500 reports about nanozymes, among which more than 1100 reports pertaining to mimicking peroxidase activity have been reached. Among them, composite nanozymes based on noble metals such as Ag, Au, and Pt and other transition metal nanoparticles with peroxidase-like activity have been successfully developed in recent years and are widely used in biomedical fields such as biosensing [16], cancer diagnosis and treatment [17,18], glucose detection [19], and antibacterial therapy [20].

In 2007, Yan et al. [21] reported that ferromagnetic nanoparticles can simulate peroxidase activity. Research on the use of nanomaterials to simulate enzyme activity developed rapidly in the following decade, and the application of transition metal nanoparticles, transition metal oxides, and their derivatives to simulate enzyme activity, as well as their nanocomposites for enzyme activity simulation, has been significantly developed. Among the transition metals, studies based on precious metals as enzyme simulators have attracted the attention of researchers in recent years due to the superior biocompatibility of Au, Ag, and Pt. In several recent studies, Zhu et al. [22] first deposited Fe_3_O_4_ nanoparticles on the surface using roughened halloysite nanotubes (RHNTs) as the substrate material. Then, after further modification, Ag nanoparticles were utilized via in situ synthesis. As a result, Ag@Fe_3_O_4_/RHNT composite nanozymes were formed. After the modification of Ag nanoparticles, sensitive colorimetric detection of hydrogen peroxide in milk and serum was achieved. Liu et al. [23] used a Zr-based metal–organic framework (Uio-66) as a matrix material and modified Au nanoparticles uniformly on the surface of the material. As a result, Au@ Uio-66 composite nanozymes were formed. Moreover, thanks to the uniform distribution of small-sized Au nanoparticles, peroxidase activity was significantly enhanced. Ultimately, effective and accurate detection of dopamine and glucose was realized. Xing et al. [24] successfully modified Pt nanoparticles on a metal–organic framework (PCN-224) centered on Zr atoms and then formed Pt@PCN-224 composite nanozymes. Moreover, the whole assay process was successfully assembled on a microfluidic chip. The constructed microfluidic biosensor can realize sensitive and specific detection of *E. coli*.

**Figure 1:**
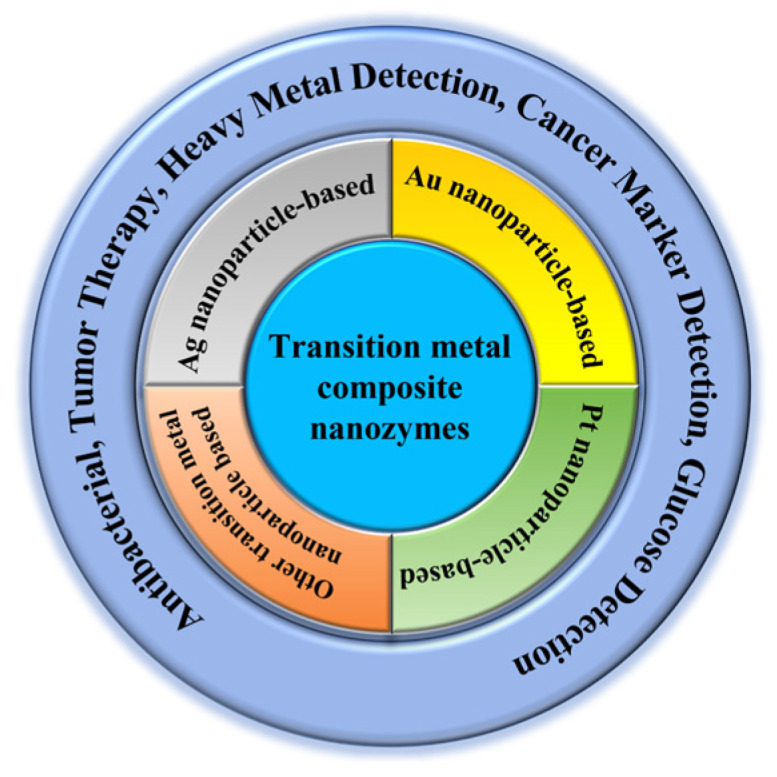
Transition-metal composite nanozymes classification and applications.

As a result, more and more researchers have been compositing two or more materials based on transition metal nanoparticles. After combining the advantages of each material, composite nanozymes with enzyme-like activity or enhanced enzyme activity are formed. The composites prepared have peroxidase-mimicking activity or enhanced peroxidase-like activity in synergy with other materials. This article focuses on composite materials developed from transition metal nanoparticles to mimic peroxidase activity and their application in the biomedical field (Figure 1).

## 2. Ag Nanoparticle-Based Composite Nanozymes

### 2.1. Ag-Based Composite Nanozymes in Disease Diagnosis

**Figure 2:**
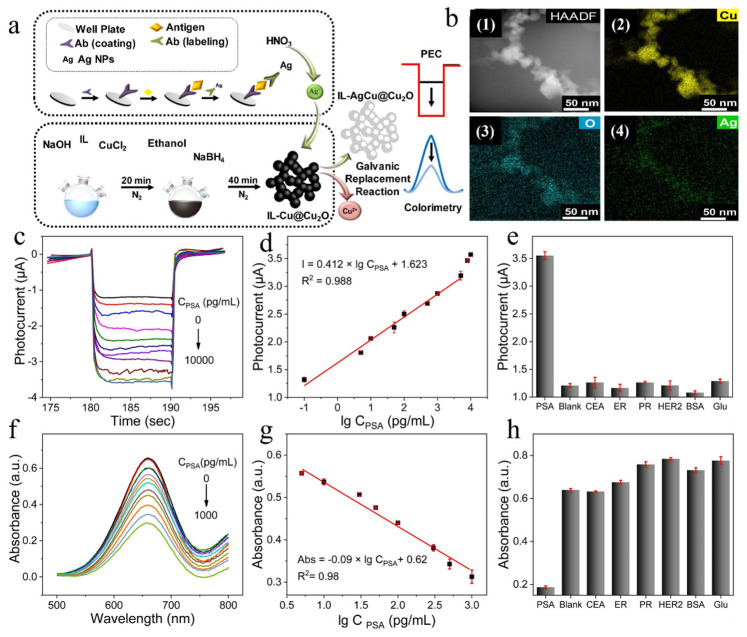
(**a**) Dual-mode biosensing platform of IL-Cu@Cu_2_O. (**b**) HAADF-STEM (1) and HAADF-STEM mapping images of Cu (2), O (3), and Ag (4) of IL-AgCu@Cu_2_O. (**c**–**e**) Photocurrents of different PSA concentrations, corresponding calibration curve and linear relation, and PSA detection selectivity of IL-Cu@Cu_2_O aerogel-based photoelectrodes, respectively. (**f**–**h**) UV–vis absorbance of different PSA concentrations, corresponding calibration curve and linear relation, and PSA detection selectivity of IL-Cu@Cu_2_O aerogel-based UV–vis absorbance, respectively. Reproduced without modification and with permission from [25] under a Creative Commons Attribution 4.0 International License (https://creativecommons.org/licenses/by-nc/4.0/, accessed on 4 September 2023).

In a relatively recent study conducted by Li et al. [25] (Figure 2a), a biosensor for the dual-mode sensitive detection of prostate-specific antigens (PSAs) was successfully constructed by using Ag nanoparticles as signal labels and amplifiers. As shown in Figure 2b, HAADF-STEM mapping images showed that Ag nanoparticles were successfully introduced and uniformly distributed on the surface of the material, and, ultimately, ionic liquid aerogels with enzymatic activity were successfully fabricated. As shown in Figure 2c.f, the sensitive detection of PSAs was carried out using both photoelectrochemical and colorimetric sensing modes. Figure 2d,g show the calibration curves in the ranges of 0.1 pg/mL to 5 ng/mL and 5 pg/mL to 1 ng/mL, respectively, as well as their good linearities (R^2^ = 0.988 and R^2^ = 0.98, respectively). Excellent selectivity for PSA detection in both photoelectrochemical and colorimetric sensing dual modes is shown in Figure 2e,h. The nanozyme prepared using this method is highly sensitive for prostate-specific antigens, which may lead to a slight decrease in the detection range due to the increased sensitivity. Therefore, there may be limitations in the application. Cancer remains one of the major diseases threatening the health of all human beings and has a low cure rate in its later stages; therefore, it is particularly important to target the pre-treatment and detection of cancer. Based on the discovery of non-coding short RNAs that can act on tumor cell development in the medical field [26], Shen et al. [27] developed an electrochemiluminescent (ECL) biosensor that was used to detect MicroRNA-155, a breast cancer marker, at very low levels. They used Ag nanoparticles coated with TiO_2_ (TiO_2_@Ag NPs) composite nanozymes that excellently mimic peroxidase activity. The reactive oxygen species produced by the catalytic decomposition of H_2_O_2_ synergistically interact with the DNA nanoframes carrying emitters to enhance the electrochemiluminescence signal output. The optical signal output gradually increases with the increasing concentration of the marker MicroRNAs. The developed nanozyme plays a crucial role in this process, which enables the ultrasensitive detection of breast cancer cell markers with a detection limit as low as 0.45 fM, which is essential for pre-tumor detection. Also, in the field of cancer, it has been found that changes in cysteine concentrations correlate greatly with the processes associated with cancer development [28], thus making the detection of cysteine important for the pre-diagnosis of cancer. Therefore, Ma et al. [29] successfully prepared a AgNPs@Fe_3_O_4_ composite nanozyme with core–shell nanostructures using a solvothermal method. On the one hand, cysteine further inhibited the oxidation of colorless o-phenylenediamine and reduced the absorbance by reacting with the reactive oxygen species generated by the AgNPs@Fe_3_O_4_ nanozyme catalyzed by H_2_O_2_. On the other hand, the adsorption of cysteine onto the surface of the nanozyme masked the active site due to electrostatic effects, resulting in decreased mimetic enzyme activity, thus reducing the oxidation of colorless o-phenylenediamine and decreasing the absorbance. The combination of the two aspects of the reaction makes it possible to use the change in absorbance to determine the concentration of cysteine for the detection of cancer markers.

The application in disease diagnosis was mainly carried out by constructing a sensor with a high sensitivity response to the concentration of hydrogen peroxide. The change in the concentration of carcinoembryonic antigen or cancer cells is signaled by the sensor. Thus, the diagnosis of the disease can be achieved.

### 2.2. Ag-Based Composite Nanozymes for Antimicrobial Applications

Since it is difficult for natural peroxidase enzymes to perform antimicrobial effects in acidic environments, there is a need to develop an acid-resistant nanozyme. Recently, Cao et al. [30] first prepared Bi_2_MoO_6_ (BMO) nanomaterials using the solvothermal method, then loaded Ag nanoparticles on BMO nanomaterials using the photo-reduction method, and finally prepared a Ag/BMO composite nanozyme. Under 1064 nm laser irradiation, due to the narrow band gap of the Ag/BMO nanozyme, electron transfer is accelerated and a large amount of reactive oxygen species is generated, which can achieve more than 99% bactericidal efficiency against bacteria in vivo. In addition, the release rate of Ag+ increases in an acidic environment, which facilitates anti-infective therapy based on the inherent antibacterial properties of Ag^+^. Therefore, the composite nanozyme developed by it works by replacing the natural enzyme and solving the nature of the natural enzyme, which is easily inactivated in the acidic environment. At the same time, the drawbacks of bacterial resistance are addressed. It provides a new idea for the development of a multifunctional nanozyme. Zhang et al. [31] utilized aminated MIL-88(Fe) nanomaterials as a substrate material and further modified Ag nanoparticles on the surface of the material, resulting in a conformational layer of NH_2_-MIL-88(Fe)-Ag composite nanozymes. The composite nanozymes were released by reacting with hydrogen peroxide, which resulted in the release of hydroxyl radicals and Ag nanoparticles. Thus, the synergistic effect of hydroxyl radicals and Ag nanoparticles can disinfect wounds and also provide excellent antimicrobial effects.

In the antibacterial process, the common point is that Ag ions are released through the reaction with hydrogen peroxide, achieving a certain antibacterial effect. The difference is that one strategy involves the production of single linear oxygen under laser irradiation, while the other involves the catalyzed decomposition of hydrogen peroxide to produce •OH. Both of them result in a synergistic effect with Ag ions to enhance the antimicrobial properties.

### 2.3. Ag-Based Composite Nanozymes for Glucose Testing

In the field of medical diagnostics and biosensing, human sweat secretions are closely related to physiological conditions in vivo. Therefore, it is a simple and practical method to detect and analyze human sweat secretions in vitro using non-invasive methods. Due to the small amount of sweat secretion, the development of a nanomaterial suitable for the ultrasensitive detection of secretion is particularly important. Guo et al. [32] prepared a Ag-Cu_2_O/reduced graphene oxide (rGO) composite nanozyme by growing Ag nanoparticles on the surface of Cu_2_O with the in situ growth method using rGO as a substrate. The substrate TMB was oxidized using •OH generated from H_2_O_2_ decomposition, and the oxidation of TMB molecules was monitored by surface-enhanced Raman scattering to realize the accurate detection of H_2_O_2_ concentration. In addition, under conditions where glucose oxidase is present, ultraprecise detection of glucose content is possible. Ultimately, the detection of glucose content in in vitro secretions enabled the differentiation between healthy people and patients with diabetes. Recently, the design, preparation, and application of multifunctional nanozymes have become popular. To have precise and effective inactivation of cancer cells while detecting glucose, Hai et al. [19] combined graphene quantum dots (GQDs) with certain reduction and stabilization capabilities. A multifunctional Ag NPs@GQDs composite nanozyme was successfully prepared by in situ growth of silver nanoparticles on a composite material consisting of GQDs and tannic acid. On the one hand, Ag NPs@GQDs nanoparticles with peroxidase-like properties can effectively kill cancer cells by degrading the high concentration of H_2_O_2_ in cancer cells to produce Ag^+^ and releasing oxidized tannins with synergistic anticancer effects. On the other hand, it can also be used as a nanoprobe to detect H_2_O_2_ produced by glucose oxidase-catalyzed glucose oxidation, thus detecting glucose in human serum samples. Therefore, the multifunctional composite nanozyme developed by it is more versatile in terms of applicability. At the same time, there is more potential for nanomaterials in clinical applications.

In the glucose detection process, the sample is oxidized by glucose oxidase to produce the corresponding hydrogen peroxide, and the concentration of hydrogen peroxide is detected. Eventually, the purpose of glucose detection is reached. The advantage of this is that the detection results are more accurate, but the detection process is more cumbersome.

## 3. Au Nanoparticle-Based Composite Nanozymes

Compared with other transition metal nanozymes, gold nanoparticles have made great progress in the field of nanozymes due to the advantage of their tunable catalytic activity [33,34]. However, gold nanoparticles also have certain shortcomings, and their poor dispersion and stability affect the enzyme mimicry activity of gold nanoparticles [35]. Therefore, combining gold nanoparticles with others to form composite nanozymes can solve these problems to a certain extent.

### 3.1. Au-Based Composite Nanozymes for Antimicrobial Applications

Dental caries and periodontitis are two common diseases among serious human health problems globally [36], and the residual bacteria that remain after treatment are a great problem for the treatment of dental diseases. To solve this problem, Cao et al. [20] developed a Au@Cu_2−x_S nanocomposite with a core–shell structure. By integrating the photothermal properties of the material and mimicking the activity of peroxidase with Beagle canine teeth as an infection model, bacteria were extracted from it and mixed and cultured with the composite nanozyme and H_2_O_2_ under laser irradiation. Its antimicrobial effect was remarkable under the synergistic effect of reactive oxygen species and localized heat. Recently, based on the reduction process using superoxide radicals to reduce tetrachloroauric acid cleaned for gold nanoparticles in the presence of water and oxygen, Cao [37] et al. developed a Au-doped Au/MoO_3−x_ composite nanozyme based on Au doping to treat methicillin-resistant Staphylococcus aureus. Compared to the undoped gold nanoparticle material, the doping of gold nanoparticles improves the photothermal conversion efficiency by about 11.3%, and the simulated peroxidase activity is enhanced by a factor of 3.6. Due to its excellent photothermal conversion efficiency and peroxidase-like activity, it generates high temperature and •OH radicals with 99.77% efficiency for bacterial inactivation. The excellent antimicrobial performance of the composites through doping based on Au nanoparticles was fully demonstrated. Recently, Li et al. [38] reduced the particle size of gold nanoparticles by modifying gold nanoparticles with bovine serum protein, which further added to the activity of multiple enzymes. They loaded small-size gold nanoparticles on molybdenum disulfide nanosheets, successfully preparing composites with mimetic superoxide dismutase, glucose oxidase, and peroxidase activities. Anchoring the composites to a hydrogel that can perform injection facilitated in vivo glucose oxidation to gluconic acid and H_2_O_2_ and oxidation of endogenous H_2_O_2_ to produce toxic •OH radicals through the inherent mimic peroxidase activity of the material, thus achieving antibacterial effects and further promoting diabetic-wound healing.

In Au-based composite nanozymes, antimicrobial therapy is mainly carried out through the decomposition of •OH produced by hydrogen peroxide decomposition, •OH, and photothermal synergy or laser irradiation, which produces reactive oxygen species and photothermal synergy.

### 3.2. Au-Based Composite Nanozymes in Glucose Detection and Cancer Cell Detection

In recent years, nanocomposites based on two-dimensional graphene-like layered structures with metal nanoparticles as loadings have received extensive attention in the field of catalysis due to their excellent catalytic activity. Cai et al. [39] prepared Au_x_Pd_100−x_ nanocomposites with a two-dimensional structure using the electrical exchange method without a reducing agent. Notably, by changing the specific surface area and the electronic structure of Pd nanosheets, the catalytic pathway was changed from the generation of •OH radicals to a rapid electron transfer process induced by phase-interface interaction. In combination with glucose oxidase, which further generates H_2_O_2_ through the oxidation of glucose, the composite with simulated peroxidase activity detects •OH radical formation, thus achieving a sensitive detection of glucose.

**Figure 3:**
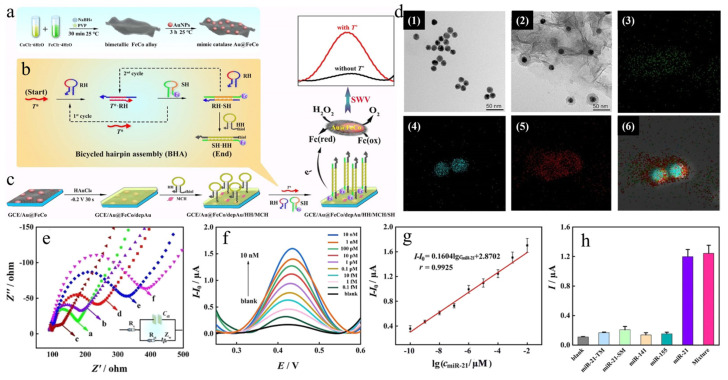
(**a**) Preparation process of Au@FeCo nanozyme. (**b**) Bicycled Hairpin Assembly (BHA) operation circuit schematic showing a specific trigger (T*) output, generated a significant amount of SH-HH biphasic product for electrode modification. (**c**) Process of constructing electrochemical biosensors for responses to T*. (**d**) TEM images of Au (1) and Au@FeCo nanozyme (2), and (3)–(6) mapping analysis of Co, Au, Fe, and Au@FeCo, respectively. (**e**) Electrochemical impedance spectra of progressively modified electrodes. (**f**) SWV responses to different concentrations of miR-21 on electrochemical biosensors. (**g**) Corresponding calibration curve and linear relation. (**h**) Specificity analysis of miR-21 using electrochemical biosensors. Reproduced without modification and with permission from [40] under a Creative Commons Attribution 4.0 International License (https://creativecommons.org/licenses/by-nc/4.0/, accessed on 4 September 2023).

With the continuous progress of science and technology, as the field of nanozyme continues to develop and advance, the development of a nanozyme that is easy to prepare and has high catalytic efficiency has been an established goal for modern researchers. In a recent study, as shown in Figure 3a, Huang and co-workers [40] prepared FeCo alloys and modified Au nanoparticles on FeCo alloys to prepare Au@FeCo composite nanozymes under room-temperature conditions. TEM images (Figure 3d) showed that the prepared Au nanoparticles were uniformly distributed on the surface of the FeCo alloy, and the mapping images of (3)–(4) in Figure 3d further demonstrated the successful preparation of the Au@FeCo nanozymes. As a result, the prepared Au@FeCo composite nanozymes possessed excellent peroxidase-like activity along with excellent electron transfer capability. Leveraging these advantages, a non-biological enzyme electrochemical biosensor was constructed (Figure 3c), and the electrode layer-by-layer modification process was confirmed using electrochemical impedance spectroscopy (Figure 3e) and tested with or without T* using square-wave voltammetry (SWV) methods on miR-21 cells within a certain concentration range. The results showed good linearity (Figure 3f,g) and satisfactory results in specific detection (Figure 3h). Although the nanozyme can be prepared conveniently and quickly with this method, the construction process of the sensor is cumbersome, which is not convenient for generalization. Since the nanozyme has favorable peroxidase mimetic activity, the nanozyme may have a broader application prospect in other fields.

### 3.3. Au-Based Composite Nanozymes as a Possible Way Forward

In general, composites with simulated peroxidase activity developed based on gold nanoparticles have good applications in both the antibacterial and disease detection fields. Recently, emerging metal-based aerogel materials have attracted a lot of attention from researchers due to the integration of the advantages of metals and aerogels, making them rich in porous structures with metal skeletons. As a typical example, Xu et al. [41] used dopamine as a gel initiator to stabilize gold nanoclusters as precursors and further dried them using CO_2_ to successfully obtain Au aerogel samples with an ultrasmall size of 3.5 nm and good catalytic stability. The aerogel exhibited good peroxidase-like activity through the oxidation of TMB. Due to its abundant metal sites and excellent biocompatibility with gold nanoparticles, it provides a new pathway for the realization of biosensing, disease detection, and cancer therapy. Li and his research group [42] successfully prepared a new Au@DNA composite nanozyme by mixing gold nanoparticles and a DNA corona. The result obtained is fivefold higher than the catalytic efficiency of the original Au nanozyme. The prepared artificial enzyme combines the advantages of nanozymes and natural enzymes, and its catalytic efficiency is higher than that of other DNA enzymes in the same oxidation reaction. It has more advantages than traditional nanozymes in terms of biocompatibility. Therefore, it has great application prospects in the field of biomedicine.

## 4. Pt Nanoparticle-Based Composite Nanozymes

Compared with other transition metals, platinum nanoparticle nanozymes not only excel in mimicking enzyme catalytic performance but also have certain advantages in biocompatibility [17,43]. However, their preparation cost is high, and they are not easy to apply on a larger scale compared to other transition metals.

### 4.1. Pt-Based Composite Nanozymes for Antibacterial Applications

In recent years, there has been great progress in the development of nanozymes based on the inherent peroxidase-like activity of Pt nanoparticles. Chen et al. [44] used ZIF-8 as a substrate to convert Pt nanoparticles into Pt monoatomics by using the strategy of reversed-heat sintering, which ultimately resulted in nanocomposites in which Pt monoatomics were homogeneously dispersed on S, P, and N co-doped substrate materials. In the absence of high concentrations of H_2_O_2_, the inactivation efficiency of five species of bacteria, including gram-negative Escherichia coli, Pseudomonas aeruginosa, Salmonella enteritidis, Klebsiella pneumoniae, and gram-positive Staphylococcus aureus, was more than 90%. The excellent peroxidase mimetic activity and broad-spectrum antimicrobial performance were demonstrated, and the preparation strategy of the nanozyme opened a new path in the rational design and optimization of the nanozyme. Fan et al. [45] developed a Pt/g-C_3_N_4_-K composite nanozyme with carbon nitride nanorods as a matrix loaded with Pt single atoms. By increasing the crystallinity and significantly enhancing the charge mobility, the desorption energy for the decomposition of OH* intermediate states by H_2_O_2_ can be significantly reduced, thus increasing the generation of •OH radicals. By virtue of the superior antimicrobial properties of •OH radicals, the bacterial inactivation efficiency was >99.9% in the presence of low concentrations of H_2_O_2_. Thus, it is demonstrated that the Pt/g-C_3_N_4_-K composite nanozyme constructed based on Pt nanoparticles exhibits superior antibacterial properties.

The homogeneous dispersion of Pt-based nanoparticles on the matrix material was enhanced by its peroxidase mimetic activity. As a result, the decomposition of hydrogen peroxide was increased to some extent, and the efficiency of •OH production was increased. Thus, the broad-spectrum antimicrobial properties were enhanced.

### 4.2. Application of Pt-Based Composite Nanozymes for Glucose and α-Glucosidase Detection

Li et al. [46] developed a composite nanozyme loaded with Pt nanoparticles on an Fe-MOF substrate. Due to the strong electronic metal–carrier interactions between Pt nanoparticles and Fe-MOF, the peroxidase mimetic activity was enhanced through a synergistic effect. The specific synergistic effect is manifested by the transfer of electrons from Pt atoms to Fe atoms, accelerating the redox cycle in divalent and third-order Fe. This demonstrates peroxidase mimetic activity, enabling colorimetric detection of glucose. The color-change response can be carried out within 2 min, and its detection limit can reach 2.3 μM, enabling sensitive and rapid detection of glucose. Similarly, Chen et al. [47] formed Pt@Fe_2_O_3_ composite nanozymes by preparing ultrathin Fe_2_O_3_ as a matrix material and modifying Pt nanoparticles. Hydrogen peroxide was generated through glucose oxidation, and then the Pt@Fe_2_O_3_ composite nanozymes catalyzed the decomposition of hydrogen peroxide to make TMB change color. Ultimately, this allowed for accurate and sensitive detection of glucose.

**Figure 4:**
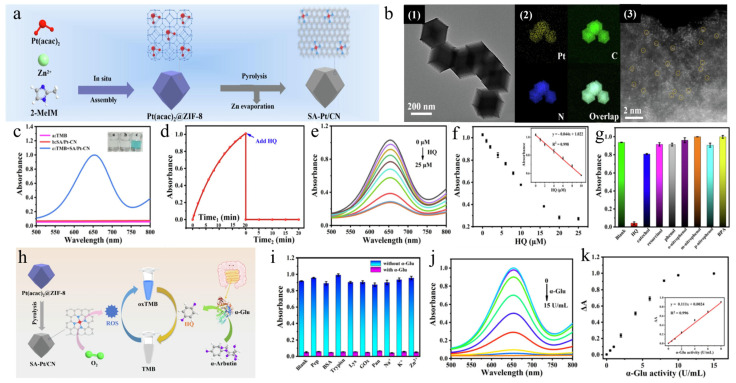
(**a**) Preparation flowchart of SA-Pt-CN. (**b**) TEM (1), EDS elemental maps (2), and enlarged (3) image of SA-Pt/CN. (**c**) Optical absorption spectra for different reaction systems. (**d**) SA-Pt/C-N-catalyzed oxidation of TMB and HQ-mediated reduction of oxTMB with time-dependent 652 nm absorbance. (**e**) Absorption spectra of different concentrations of HQ in SA-Pt/CN-TMB-HQ solutions. (**f**) Absorbance at 652 nm for different HQ concentrations. A linearity plot is shown in the inset. (**g**) HQ specificity analysis. (**h**) α-glucosidase detection schematic diagram. (**i**) Comparison of absorbance in the presence and absence of α-Glu. (**j**) Absorption spectra of different concentrations of α-glucosidase in SA-Pt/CN-TMB- α-glucosidase solutions. (**k**) Absorbance at 652 nm for different HQ concentrations. A linearity plot is shown in the inset. Reproduced without modification and with permission from [48] under a Creative Commons Attribution 4.0 International License (https://creativecommons.org/licenses/by-nc/4.0/, accessed on 4 September 2023).

The zinc-based zeolitic imidazolium framework (ZIF-8) material has an abundant nitrogen-doped porous carbon structure after pyrolysis, which is favorable for the transport of catalytic substrates and the loading of metal nanoparticles. Based on this, Chen and his team, Ref. [48], assembled Pt(acac)_2_@ZIF-8 in situ by using ZIF-8 as a sacrificial template to generate Pt(acac)_2_@ZIF-8. They further pyrolyzed it to generate SA-Pt/CN composite nanozymes (Figure 4a), and the pyrolyzed Pt single atoms could be dispersed uniformly in the material (Figure 4b). By taking advantage of the fact that hydroquinone (HQ) can effectively affect the oxidation effect of TMB, causing a significant decrease in absorbance at 652 nm (Figure 4d), a detection system for hydroquinone was constructed. The experimental results are shown in Figure 4e,f. The hydroquinone concentration showed excellent linearity (R^2^ = 0.998) within the concentration range of 0–10 μM, demonstrating excellent specificity. Moreover, for the concentration of α-glucosidase, the experimental flowchart is shown in Figure 4h. α-Arbutin reacts with α-glucosidase to generate hydroquinone, which reduces the absorbance at 652 nm, thus realizing the detection of α-glucosidase. As shown in Figure 4i, there was a significant difference in absorbance with and without the presence of α-glucosidase. The corresponding experimental results are shown in Figure 4j,k. The concentration of α-glucosidase showed good linearity in the range of 0.01–8 U/mL (R^2^ = 0.996). The use of this method provides a novel and simpler strategy for the constructed Pt atom composite-based nanozyme. Moreover, the ability to realize the detection of hydroquinone and α-glucosidase improves the application of this nanozyme.

In Pt-based composite nanozymes for glucose detection, hydrogen peroxide is produced by the oxidation of glucose via glucose oxidase. The glucose content is determined indirectly by measuring the hydrogen peroxide content. Similarly, the determination of glucosidase is also indirect.

### 4.3. Pt-Based Composite Nanozymes for Tumor Cell Inactivation and Mercury Ion Detection

In the recent past, novel transition metal carbide/nitride nanosheets (MXenes), with their good biocompatibility and electrical conductivity, have gained special attention in the biomedical field [49]. Utilizing advantages such as good biocompatibility, Shi et al. [50] developed a precise construction method for nanocomposites based on Ti_3_C_2_Tx MXenes using DNA-encoded in situ growth of platinum atoms. The peroxidase mimetic activity of the nanocomposites was reduced by Pt nanoparticles with reduced mercury ions. By regulating the concentration of mercury ions, a colorimetric assay was applied to ultimately determine the concentration of Hg^2+^ in the samples. Similarly, Zhu et al. [51] successfully developed a nanocomposite with simulated peroxidase activity by modifying Pt nanoparticles and using Ti_3_C_2_ nanosheets as a substrate material. The synergistic effect of the superior photothermal effect of Ti_3_C_2_ nanosheets under near-infrared light conditions led to enhanced peroxidase activity of Pt nanoparticles and endogenous H_2_O_2_ decomposition to produce toxic •OH radicals, thus allowing cell death and apoptosis. Sheng et al. [52] modified Pt nanoparticles on the surface of the material by in situ synthesis using CuS as a substrate material to form Pt@CuS composite nanozymes. Subsequently, the biocompatibility and photodynamic therapeutic effects were increased by the incorporation of photosensitizers and biomaterials. Leveraging the excellent catalytic properties of platinum nanoparticles, the efficiency of H_2_O_2_ decomposition to generate oxygen was increased. The generation of ^1^O_2_ was effectively increased, producing better results for tumor therapy.

Pt-based composite nanozymes are used in tumor cell therapy by virtue of the excellent peroxidase mimetic activity of Pt nanoparticles. Under the synergistic effect of laser irradiation conditions, they show high inhibition and inactivation efficiency against tumor cells. Therefore, their application in the clinical treatment of tumor cells has great potential.

## 5. Other Transition-Metal-Nanoparticle-Based Composite Nanozymes

### 5.1. Advances in Other Transition-Metal Composite Nanozymes for Tumor/Cancer Cell Applications

**Figure 5:**
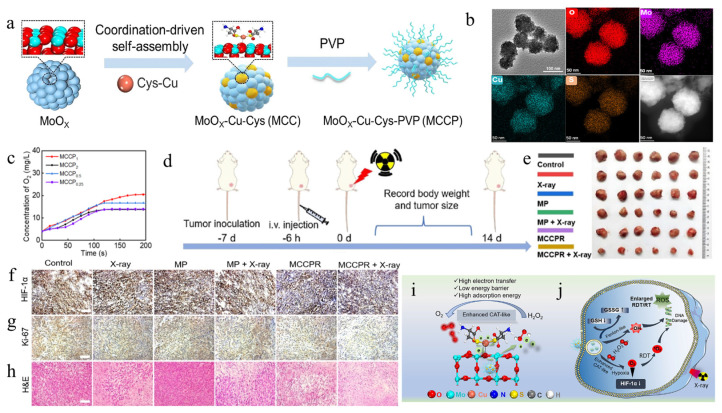
(**a**) Schematic process diagram for the construction of Cu single-atom MCCP composite nanozymes. (**b**) TEM images of MCCP SAzymes, elemental mapping for Cu, Mo, S, and O, and corresponding HAADF-STEM images of MCC. (**c**) O_2_ concentrations for MCCP samples with various Cu loading rates in the presence of 1 mM H_2_O_2_ (pH = 5.0; temperature: 25 °C). (**d**) Schematic diagram of 4T1 tumor model establishment and treatment protocol after drug administration. (**e**) Representative photograph of the tumor on day 28 after treatment. (**f**) HIF-1α, (**g**) Ki-67, and (**h**) H&E coloring of tumors after various treatments. (**i**) Schematic diagram of MCCP structure and electron transfer process with enhanced catalase-like activity to increase oxygen production. (**j**) MCCP acts as tumor-specific nanozymes with enhanced catalytic activity, thereby enhancing radiosensitization of radiodynamics. Reproduced without modification and with permission from [53] under a Creative Commons Attribution 4.0 International License (https://creativecommons.org/licenses/by-nc/4.0/, accessed on 4 September 2023).

Zhao et al. [53] proposed a self-assembly strategy for transition metal ions, as shown in Figure 5a, and successfully prepared the composite MoOx-Cu-Cys nanoconjugate (MCC) based on Cu single atoms, followed by the modification of polyvinylpyrrolidone (PVP) to improve biocompatibility, which ultimately resulted in the formation of the composite MoOx-Cu-Cys-PVP nanoconjugate (MCCP). As shown in Figure 5b, the synthesized MCC is uniformly spherical, and O, Mo, P, and S elements are uniformly distributed in the material. Putting the ratio of copper nanozyme atoms at 10 w% allows for optimal catalytic efficiency and the strongest oxygen production capacity of MCCP (Figure 5c). The catalytic efficiency of the final prepared nanozymes was enhanced by a factor of 14.3 compared to natural catalase. Before the antitumor treatment, the surface of MCCP was modified with a targeting group (called MCCPR). This modification was used to improve the precise recognition ability of the nanozyme for tumor cells. Figure 5d shows the experimental flow of MCCPR antitumor treatment. By analyzing the varying experimental conditions, it can be seen that the tumor cells were significantly inhibited by MCCPR+X-ray (Figure 5e). Under the conditions of MCCPR+X-ray treatment, the expression of HIF-1α was decreased, probably due to the higher catalysis activity of MCCPR for mimicking catalase (Figure 5f). Ki-67 immunohistochemistry demonstrated that the value-added of the tumor cells was obviously inhibited by the inhibitory effect, and the cells of the H&E-stained tumor sections were obviously apoptotic. In summary, the transition-metal composite nanozyme was prepared through the self-assembly effect on transition metals, which saves costs and improves convenience to a certain extent. Although the cascade reaction of MCCP+X-ray can effectively inhibit tumor cell growth and inactivate tumor cells, the in vivo operation is cumbersome and needs to be further optimized to improve convenience.

To address the problem of the tumor microenvironment, where photodynamic therapy is difficult due to hypoxia, Sang et al. [54] developed a novel composite nanozyme (Ni_3_S_2_Cu_1.8_S) with a Z-type nanoheterostructure. Hyaluronic acid, which has the ability to specifically recognize cancer cells, was modified on the surface of the material, and through the mimic peroxidase activity possessed by their composite nanozyme, it could decompose intracellular H_2_O_2_ to produce •OH and oxygen to compensate for the hypoxic environment inside the tumor cells. Since the material forms a narrow band gap favorable for electron–hole separation, under NIR laser irradiation, photoexcited holes directly oxidize H_2_O to O_2_, and photoexcited electrons can further transform O_2_ into toxic reactive oxygen species for effective tumor cell inactivation. Meanwhile, its composite material nanozyme can be degraded and eliminated by biological metabolic activities, will not be residual in the body, and will not become harmful to the organism itself.

To improve tumor cells’ growth inhibition and targeting therapeutic abilities, Liu et al. [55] developed CuMnOx nanoparticles stabilized by hyaluronic acid and further loaded with indocyanine green, thus forming HA-CuMnOx@ICG composite nanozymes (CMOI NCs). CMOI NCs, modified with hyaluronic acid, had specific target recognition in HeLa cells. Under light conditions, they could effectively generate reactive oxygen species and carry out localized photothermal treatment, which could effectively kill HeLa cells under the dual effects of reactive oxygen species and localized heat. Similarly, Alizadeh et al. [56] developed a Co(OH)_2_/FeOOH/WO_3_ composite nanozyme with high stability and a large surface area through a hydrothermal reaction. They achieved precise recognition of cancer cells through the modification of the HeLa cell-targeting ligand folate and the oxidation of TMB due to its superior mimetic peroxidase activity. Selective recognition of HeLa cells was achieved within the range of 50–5 × 10^4^ cells/mL (according to the color change) and combined with methylene blue (MB) for successful in vitro cancer cell inactivation under light conditions.

For other transition technology nanocomposite nanozymes, more attention has been given to the field of inhibition and inactivation of tumor cells. Through the decomposition of hydrogen peroxide by nanozymes, a large amount of oxygen is produced inside the cell, and under laser irradiation, the cellular oxygen is converted into reactive oxygen species. Thus, the tumor cells can be effectively inhibited and inactivated.

### 5.2. Other Transition-Metal Composite Nanozymes in Other Applications

The application of bionics is extremely common in life and offers numerous contributions to the development of nanozymes. Among them, Liu et al. [57] developed a novel nanocomposite with peroxidase-like activity by mimicking natural enzymes. Using human serum proteins and polydopamine as the basic skeleton structure and Fe^2+^ and Fe^3+^ as the active centers, a novel nanocomposite with peroxidase-like activity and enzyme-like structure to improve selectivity and activity was constructed. The conventional colorimetric method was also used for the in situ detection of H_2_O_2_ produced by living cells and the in vitro detection of H_2_O_2_, and a sensitive detection was finally achieved.

While exploring the reason why transition metal oxides can mimic peroxidase activity, Yuan et al. [58] found that the generation of •OH radicals is specifically due to the redox reaction between surface metal cations and H_2_O_2_. The generated •OH can further reduce the oxidized metal cations and promote the decomposition of H_2_O_2_, thus generating •OH radicals. The generation of •OH radicals was identified as a rate-limiting step. It provides some schematic support for the future synthesis of composite nanozymes with transition metal oxides.

## 6. Conclusions

For glucose detection, Ag-, Au-, and Pt-based composite nanozymes were used in a cascade reaction. The detection is cumbersome, but the Pt-based material shows good sensitivity by virtue of its excellent peroxidase mimetic activity. Thanks to their antimicrobial properties, Ag-based composite nanozymes can inhibit and inactivate bacteria through the synergistic effect of Ag ions and •OH. While Au- and Pt-based composite nanozymes can only be antimicrobial through •OH produced by their own powerful peroxidase mimicry activity, they can also be synergistically antimicrobial when exposed to laser irradiation. Finally, for the application of tumor cell inhibition and killing, Pt-based nanomaterials and other transition metal nanomaterials almost consistently adopt the laser irradiation strategy of converting oxygen into reactive oxygen species. The synergistic effect of laser irradiation and catalytic hydrogen peroxide decomposition is used for tumor treatment.

This paper focuses on the development of composite nanoparticles based on transition metal nanoparticles for biomedical applications that has taken place in recent years. Compared with non-precious metal transition metals, the development of nanoparticles based on precious metal nanoparticles is still a hot research topic due to their excellent mimetic enzyme activity and good biocompatibility. Due to the high costs, it is still an urgent problem to develop non-precious metal composite nanoparticles with good biocompatibility, thoroughly elaborate the mechanism of action of the material, and reduce the production costs while maintaining high mimetic enzyme activity. Compared to precious metal transition-metal composite nanozymes, other transition metal nanozymes, although less expensive to prepare, have similarly lower mimetic enzyme activity. They can be used to improve simulated enzyme activity through synergistic interactions between the metals, but progress has been slower. In conclusion, the realization of transition-metal composite nanozymes is still a challenge, and the progress of their application is still unclear.

## Data Availability

The data presented in this study are available on request from the corresponding authors.

## References

[1] Gulcin I., Taslimi P., Aygun A., Sadeghian N., Bastem E., Kufrevioglu O.I., Turkan F., Sen F. (2018). Antidiabetic and antiparasitic potentials: Inhibition effects of some natural antioxidant compounds on alpha-glycosidase, alpha-amylase and human glutathione S-transferase enzymes. Int. J. Biol. Macromol..

[2] Zou Y., Li H., Graham E.T., Deik A.A., Eaton J.K., Wang W., Sandoval-Gomez G., Clish C.B., Doench J.G., Schreiber S.L. (2020). Cytochrome P450 oxidoreductase contributes tophospholipid peroxidation in ferroptosis. Nat. Chem. Biol..

[3] Song Z., Cai Y., Lao X., Wang X., Lin X., Cui Y., Kalavagunta P.K., Liao J., Jin L., Shang J. (2019). Taxonomic profiling and populational patterns of bacterial bile salt hydrolase (BSH) genes based on worldwide human gut microbiome. Microbiome.

[4] Paul B.D., Sbodio J.I., Xu R., Vandiver M.S., Cha J.Y., Snowman A.M., Snyder S.H. (2014). Cystathionine γ-Lyase deficiency mediates neurodegeneration in huntington’s disease. Nature.

[5] Fu M., Zhang W., Wu L., Yang G., Li H., Wang R. (2012). Hydrogen sulfide (H_2_S) metabolism in mitochondria and its regulatory role in energy production. Proc. Natl. Acad. Sci. USA.

[6] Liu Z., Pan Q., Ding S., Qian J., Xu F., Zhou J., Cen S., Guo F., Liang C. (2013). The interferon-inducible MxB protein inhibits HIV-1 infection. Cell Host Microbe.

[7] Nguyen G.K.T., Wang S., Qiu Y., Hemu X., Lian Y., Tam J.P. (2014). Butelase 1 is an Asx-specific ligase enabling peptide macrocyclization and synthesis. Nat. Chem. Biol..

[8] Zhang S., Zhao W., Zeng J., He Z., Wang X., Zhu Z., Hu R., Liu C., Wang Q. (2023). Wearable non-invasive glucose sensors based on metallic nanomaterials. Mater. Today Bio.

[9] Nasrollahzadeh M., Sajjadi M., Iravani S., Varma R.S. (2021). Carbon-based sustainable nanomaterials for water treatment: State-of-art and future perspectives. Chemosphere.

[10] Liu X., Zhao Y., Xu Y., Liu C. (2023). Synthesis of gamma-cyclodextrin-reduced Fe(III) nanoparticles with peroxidase-like catalytic activity for bacteriostasis of food. Nano Lett..

[11] Liang M., Yan X. (2019). Nanozymes: From new concepts, mechanisms, and standards to applications. Acc. Chem. Res..

[12] Wu F., Chen H., Liu R., Suo Y., Li Q., Zhang Y., Liu H., Cheng Z., Chang Y. (2022). Modulation of the tumor immune microenvironment by Bi_2_Te_3_-Au/Pd-Based theranostic nanocatalysts enables efficient cancer therapy. Adv. Healthc. Mater..

[13] Ding Y., Wang Z., Zhang Z., Zhao Y., Yang S., Zhang Y., Yao S., Wang S., Huang T., Zhang Y. (2022). Oxygen vacancy-engineered BaTiO_3_ nanoparticles for synergistic cancer photothermal, photodynamic, and catalytic therapy. Nano Res..

[14] Zhang Y., Wang F., Liu C., Wang Z., Kang L., Huang Y., Dong K., Ren J., Qu X. (2018). Nanozyme decorated metal-organic frameworks for enhanced photodynamic therapy. ACS Nano.

[15] Jiao L., Wu J., Zhong H., Zhang Y., Xu W., Wu Y., Chen Y., Yan H., Zhang Q., Gu W. (2020). Densely isolated FeN_4_ sites for peroxidase mimicking. ACS Catal..

[16] Sharifi M., Hosseinali S.H., Yousefvand P., Salihi A., Shekha M.S., Aziz F.M., JouyaTalaei A., Hasan A., Falahati M. (2020). Gold nanozyme: Biosensing and therapeutic activities. Mater. Sci. Eng. C Mater. Biol. Appl..

[17] Liu C., Xing J., Akakuru O.U., Luo L., Sun S., Zou R., Yu Z., Fang Q., Wu A. (2019). Nanozymes-engineered metal–organic frameworks for catalytic cascades-enhanced synergistic cancer therapy. Nano Lett..

[18] Ou D., Sun D., Lin X., Liang Z., Zhong Y., Chen Z. (2019). A dual-aptamer-based biosensor for specific detection of breast cancer biomarker HER2 via flower-like nanozymes and DNA nanostructures. J. Mater. Chem. B.

[19] Hai X., Li Y., Yu K., Yue S., Li Y., Song W., Bi S., Zhang X. (2021). Synergistic in-situ growth of silver nanoparticles with nanozyme activity for dual-mode biosensing and cancer theranostics. Chin. Chem. Lett..

[20] Cao J., Sun Q., Shen A.-G., Fan B., Hu J.-M. (2021). Nano Au@Cu_2−x_S with near-infrared photothermal and peroxidase catalytic activities redefines efficient antibiofilm-oriented root canal therapy. Chem. Eng. J..

[21] Gao L., Zhuang J., Nie L., Zhang J., Zhang Y., Gu N., Wang T., Feng J., Yang D., Perrett S. (2007). Intrinsic peroxidase-like activity of ferromagnetic nanoparticles. Nat. Nanotechnol..

[22] Zhu X., Song P., Hou S., Zhao H., Gao Y., Wu T., Liu Q. (2023). Synthesis of Ag nanoparticles supported on magnetic halloysite nanozyme for detection of H_2_O_2_ in milk and serum. Appl. Clay Sci..

[23] Liu Q., Wang H., Yang Q., Tong Y., He W. (2022). Metal–organic frameworks loaded Au nanozymes with enhanced peroxidase-like activity for multi-targeted biodetection. Mater. Adv..

[24] Xing G., Shang Y., Ai J., Lin H., Wu Z., Zhang Q., Lin J.-M., Pu Q., Lin L. (2023). Nanozyme-mediated catalytic signal amplification for microfluidic biosensing of foodborne bacteria. Anal. Chem..

[25] Li J., Wang H., Liu M., Qin Y., Tan R., Hu L., Gu W., Zhu C. (2023). Galvanic replacement reaction-regulated photoelectric response and enzyme-mimicking property of ionic liquid functionalized Cu@Cu_2_O aerogels for dual-mode immunoassay. Chem. Eng. J..

[26] Lin S., Gregory R.I. (2015). MicroRNA biogenesis pathways in cancer. Nat. Rev. Cancer.

[27] Shen B., Wu Q., Fan Y., Wu H., Li X., Zhao X., Wang Y., Ding S., Zhang J. (2022). TiO_2_@Ag nanozyme enhanced electrochemiluminescent biosensor coupled with DNA nanoframework-carried emitters and enzyme-assisted target recycling amplification for ultrasensitive detection of microRNA. Chem. Eng. J..

[28] Lin J., Lee I.M., Song Y., Cook N.R., Selhub J., Manson J.E., Buring J.E., Zhang S.M. (2010). Plasma homocysteine and cysteine and risk of breast cancer in women. Cancer Res..

[29] Mazhani M., Alula M.T., Murape D. (2020). Development of a cysteine sensor based on the peroxidase-like activity of AgNPs@ Fe_3_O_4_ core-shell nanostructures. Anal. Chim. Acta.

[30] Cao C., Zhang T., Yang N., Niu X., Zhou Z., Wang J., Yang D., Chen P., Zhong L., Dong X. (2022). POD Nanozyme optimized by charge separation engineering for light/pH activated bacteria catalytic/photodynamic therapy. Signal Transduct. Target. Ther..

[31] Zhang W., Ren X., Shi S., Li M., Liu L., Han X., Zhu W., Yue T., Sun J., Wang J. (2020). Ionic silver-infused peroxidase-like metal–organic frameworks as versatile “antibiotic” for enhanced bacterial elimination. Nanoscale.

[32] Guo Y., Wang H., Ma X., Jin J., Ji W., Wang X., Song W., Zhao B., He C. (2017). Fabrication of Ag-Cu_2_O/reduced graphene oxide nanocomposites as surface-enhanced raman scattering substrates for in situ monitoring of peroxidase-like catalytic reaction and biosensing. ACS Appl. Mater. Interfaces.

[33] Cheng X., Sun R., Yin L., Chai Z., Shi H., Gao M. (2017). Light-triggered assembly of gold nanoparticles for photothermal therapy and photoacoustic imaging of tumors in vivo. Adv. Mater..

[34] Lin G., Xian L., Zhou X., Wang S., Shah Z.H., Edwards S.A., Gao Y. (2020). Design and one-pot synthesis of capsid-like gold colloids with tunable surface roughness and their enhanced sensing and catalytic performances. ACS Appl. Mater. Interfaces.

[35] Dhakshinamoorthy A., Asiri A.M., Garcia H. (2017). Metal organic frameworks as versatile hosts of au nanoparticles in heterogeneous catalysis. ACS Catal..

[36] Ribeiro J.S., Daghrery A., Dubey N., Li C., Mei L., Fenno J.C., Schwendeman A., Aytac Z., Bottino M.C. (2020). Hybrid antimicrobial hydrogel as injectable therapeutics for oral infection ablation. Biomacromolecules.

[37] Cao M., Chang Z., Tan J., Wang X., Zhang P., Lin S., Liu J., Li A. (2022). Superoxide radical-mediated self-synthesized Au/MOO_3-x_ hybrids with enhanced peroxidase-like activity and photothermal effect for anti-mrsa therapy. ACS Appl. Mater. Interfaces.

[38] Li Y., Fu R., Duan Z., Zhu C., Fan D. (2022). Injectable hydrogel based on defect-rich multi-nanozymes for diabetic wound healing via an oxygen self-supplying cascade reaction. Small.

[39] Cai S., Fu Z., Xiao W., Xiong Y., Wang C., Yang R. (2020). Zero-dimensional/two-dimensional Au_x_Pd_100−x_ nanocomposites with enhanced nanozyme catalysis for sensitive glucose detection. ACS Appl. Mater. Interfaces.

[40] Huang W., Yang C., Gao J., Ye J., Yuan R., Xu W. (2023). Cooperative amplification of Au@FeCo as mimetic catalytic nanozymes and bicycled hairpin assembly for ultrasensitive electrochemical biosensing. Anal. Chem..

[41] Xu J., Sun F., Li Q., Yuan H., Ma F., Wen D., Shang L. (2022). Ultrasmall gold nanoclusters-enabled fabrication of ultrafine gold aerogels as novel self-supported nanozymes. Small.

[42] Zuo L., Ren K., Guo X., Pokhrel P., Pokhrel B., Hossain M.A., Chen Z.X., Mao H., Shen H. (2023). Amalgamation of DNAzymes and nanozymes in a coronazyme. J. Am. Chem. Soc..

[43] Wang X., Wei G., Liu W., Zhang Y., Zhu C., Sun Q., Zhang M., Wei H. (2023). Platinum-Nickel nanoparticles with enhanced oxidase-like activity for total antioxidant capacity bioassay. Anal. Chem..

[44] Chen Y., Wang P., Hao H., Hong J., Li H., Ji S., Li A., Gao R., Dong J., Han X. (2021). Thermal atomization of platinum nanoparticles into single atoms: An effective strategy for engineering high-performance nanozymes. J. Am. Chem. Soc..

[45] Fan Y., Gan X., Zhao H., Zeng Z., You W., Quan X. (2022). Multiple application of SAzyme based on carbon nitride nanorod-supported Pt single-atom for H_2_O_2_ detection, antibiotic detection and antibacterial therapy. Chem. Eng. J..

[46] Li J., Zhao J., Li S., Chen Y., Lv W., Zhang J., Zhang L., Zhang Z., Lu X. (2021). Synergistic effect enhances the peroxidase-like activity in platinum nanoparticle-supported metal—Organic framework hybrid nanozymes for ultrasensitive detection of glucose. Nano Res..

[47] Chen Y., Yuchi Q., Li T., Yang G., Miao J., Huang C., Liu J., Li A., Qin Y., Zhang L. (2020). Precise engineering of ultra-thin Fe_2_O_3_ decorated Pt-based nanozymes via atomic layer deposition to switch off undesired activity for enhanced sensing performance. Sens. Actuators B.

[48] Kang G., Liu W., Liu F., Li Z., Dong X., Chen C., Lu Y. (2022). Single-atom Pt catalysts as oxidase mimic for p-benzoquinone and α-glucosidase activity detection. Chem. Eng. J..

[49] Tang M., Yue Z., Li J., Sun T., Chen C. (2023). Nanozymes based on Mxene nanosheets decorated with pt nanoparticles for hyperthermal-enhanced cascade catalytic therapy of tumors. ACS Appl. Nano Mater..

[50] Shi Y., Liu Z., Liu R., Wu R., Zhang J. (2022). DNA-encoded MXene-Pt nanozyme for enhanced colorimetric sensing of mercury ions. Chem. Eng. J..

[51] Zhu Y., Wang Z., Zhao R., Zhou Y., Feng L., Gai S., Yang P. (2022). Pt decorated Ti_3_C_2_T_x_ MXene with NIR-II light amplified nanozyme catalytic activity for efficient phototheranostics. ACS Nano.

[52] Sheng Y., Ren Q., Tao C., Wen M., Qu P., Yu N., Li M., Chen Z., Xie X. (2023). Construction of PEGylated chlorin e6@CuS-Pt theranostic nanoplatforms for nanozymes-enhanced photodynamic-photothermal therapy. J. Colloid Interface Sci..

[53] Zhou J., Xu D., Tian G., He Q., Zhang X., Liao J., Mei L., Chen L., Gao L., Zhao L. (2023). Coordination-driven self-assembly strategy-activated Cu single-atom nanozymes for catalytic tumor-specific therapy. J. Am. Chem. Soc..

[54] Sang D., Wang K., Sun X., Wang Y., Lin H., Jia R., Qu F. (2021). NIR-Driven intracellular photocatalytic O_2_ tvolution on Z-Scheme Ni_3_S_2_/Cu_1.8_S@HA for hypoxic tumor therapy. ACS Appl. Mater. Interfaces.

[55] Lv W., Cao M., Liu J., Hei Y., Bai J. (2021). Tumor microenvironment-responsive nanozymes achieve photothermal-enhanced multiple catalysis against tumor hypoxia. Acta Biomater..

[56] Alizadeh N., Salimi A., Sham T.-K., Bazylewski P., Fanchini G., Fathi F., Soleimani F. (2021). Hierarchical Co(OH)_2_/FeOOH/WO_3_ ternary nanoflowers as a dual-function enzyme with pH-switchable peroxidase and catalase mimic activities for cancer cell detection and enhanced photodynamic therapy. Chem. Eng. J..

[57] Liu X., Qin J., Zhang X., Zou L., Yang X., Wang Q., Zheng Y., Mei W., Wang K. (2020). The mechanisms of HSA@PDA/Fe nanocomposites with enhanced nanozyme activity and their application in intracellular H_2_O_2_ detection. Nanoscale.

[58] Yuan B., Chou H.L., Peng Y.K. (2021). Disclosing the origin of transition metal oxides as peroxidase (and catalase) mimetics. ACS Appl. Mater. Interfaces.

